# Effects of Oils and Essential Oils from Seeds of *Zanthoxylum schinifolium* against Foodborne Viral Surrogates

**DOI:** 10.1155/2014/135797

**Published:** 2014-12-18

**Authors:** Mi Oh, Mi Sook Chung

**Affiliations:** Department of Food and Nutrition, Duksung Women's University, Seoul 132-714, Republic of Korea

## Abstract

Human noroviruses are the most frequent cause of foodborne viral disease and are responsible for the vast majority of nonbacterial gastroenteritis. However, no specific therapies are available for the efficient control or prevention of foodborne viral disease. Here, we determined the antiviral activities of oils from seeds of *Zanthoxylum schinifolium * (ZSO) against foodborne viral surrogates, feline calicivirus-F9 (FCV-F9), and murine norovirus-1 (MNV-1), using plaque assay. Time-of-addition experiments were designed to determine the antiviral mechanism of action of ZSO against the surrogates. Maximal antiviral effect was observed upon pretreatment of FCV-F9 or MNV-1 with ZSO, which comprised oleic acid, linoleic acid, palmitic acid, and linolenic acid as the major fatty acids. FCV-F9 was more sensitive to ZSO than MNV-1, and the 50% effective concentration of ZSO against pretreatment of FCV-F9 was 0.0007%. However, essential oils from *Z. schinifolium* (ZSE), which comprised 42% estragole, showed no inhibitory effects against FCV-F9 and MNV-1. These results suggest that the inhibitory activities of ZSO were exerted by direct interaction of FCV-F9 or MNV-1 virion with ZSO, which may be a food material candidate for control of foodborne viral disease.

## 1. Introduction

Human norovirus, a member of the Caliciviridae family, is responsible for approximately 90% of epidemic nonbacterial outbreaks of gastroenteritis globally in people of all ages [1, 2]. It is highly contagious, often leading to large outbreaks in institutional food services such as schools, hospitals, childcare centers, nursing homes, and military camps, in which young children, the elderly, soldiers, and immunosuppressed patients are high-risk populations [3]. Norovirus genus is classified into five genogroups, which can be further divided into different genotypes. Genogroup II is the most prevalent, and genogroup II genotype 4 (G II-4) is responsible for most infections worldwide [4]. However, human norovirus has been relatively understudied due to lack of a suitable* in vitro* culture system. Feline calicivirus (FCV) and murine norovirus-1 (MNV-1) from STAT1-deficient (STAT1^−/−^) mice have been used as surrogates to elucidate norovirus biology and replication [5–7]. However, no specific therapies are yet available to efficiently control or prevent foodborne noroviral disease.

Seeds and pericarp of* Zanthoxylum schinifolium*, which belongs to the Rutaceae family, are widely consumed in Korea, China, and Japan as a spice.* Z*.* schinifolium* has been used in folk medicine for treatment of vomiting, diarrhea, and abdominal pain [8]. The pericarp of* Z*.* schinifolium* is also used as an antimicrobial and antioxidant. These biological functions are due to pericarp essential oils [9]. Essential oils, which are secondary metabolites of aromatic plants, have a distinct odor and are extracted from various parts of plants by hydrodistillation. Their main constituents, for example, terpenes, alcohols, aldehydes, and esters, are responsible for their biological properties such as antimicrobial activity.

Essential oils have been applied as flavoring agents to foods such as meat and have shown a wide spectrum of antimicrobial activity against several foodborne pathogens and spoilage bacteria, both* in vitro* and in food matrices [10]. Several research groups have demonstrated antiviral activities of essential oils against FCV [11], MNV-1 [12], herpes simplex virus type 1 (HSV-1) [13], HSV-2 [14], dengue virus type 2, and Junin virus [15]. Even though essential oils from* Z. schinifolium *have been demonstrated as having antibacterial activities against* Staphylococcus epidermidis* and* Bacillus subtilis *[16], no information is available on the antiviral effects of oils and essential oils from seeds of* Z*.* schinifolium* against FCV-F9 and MNV-1. In the present study, antiviral activities of oils (ZSO) and essential oils (ZSE) from seeds of* Z. schinifolium* against FCV-F9 and MNV-1 were analyzed using cytopathic effect assay and plaque assay. Maximal antiviral effect was observed upon pretreatment of FCV-F9 or MNV-1 with ZSO.

## 2. Materials and Methods

### 2.1. Viruses, Cell Lines, and* Z. schinifolium* Seed Oils (ZSO)

RAW 264.7 and Crandell Reese Feline kidney (CRFK) cells and FCV-F9 were obtained from ATCC (Manassas, VA, USA). MNV-1 was kindly provided by Dr. Herbert Virgin, Washington University School of Medicine, St Louis, USA. ZSO were purchased from Sanchonara (Jinan-gun, Jeollabuk-do, Korea). Before ZSO preparation,* Z*.* schinifolium *seeds under investigation were identified by staff members of Specialization Crop Research Institute, Jeollabuk-do Agricultural Research & Extension Services, Korea. Dried seeds of* Z*.* schinifolium *were ground by a roll-mill and steamed for 30 min, after which ZSO were extracted using an oil press. The yield of ZSO was 80.1% (v/w) (Table 1).

### 2.2. Extraction and Identification of Essential Oils from* Z. schinifolium* (ZSE)

ZSE were extracted from 300 g of dried and crushed seeds of* Z*.* schinifolium* by hydrodistillation for 2 h using a Clevenger-type apparatus (EssenLab-plus; Hanil Labtech Ltd., Incheon, Korea) [17]. The yield of ZSE, which were dried over anhydrous sodium sulfate, was 0.13% (v/w). GC/MS (Agilent 6890 GC/5973 MSD; Agilent Co., Palo Alto, CA, USA) was used to analyze essential oils with HP-5MS column chromatography (30 m length × 0.25 mm i.d. × 0.25 *μ*m film thickness; Agilent Co.). Injection volume was 1 *μ*L, and the flow rate of helium as a carrier gas was 1.0 mL/min. The oven temperature was held constant at 40°C for 5 min, increased to 220°C at a rate of 5°C/min, and then held constant at 220°C for an additional 5 min. The temperatures of the injector and detector were 200 and 250°C, respectively. The MS was operated in electron ionization mode at 70 eV, scanning masses from 33 to 330 m/z. Identification of peaks on the chromatogram was based on mass spectra and retention indices (RIs). Mass spectra for unknowns were compared with those in an online computer library (Wiley 275; Agilent Co.). The RIs of chromatographic peaks, determined using a series of alkanes as external references (hydrocarbon C5-C30; Aldrich Co, Milwaukee WI, USA), were compared with those in the published literature [18].

### 2.3. Cytotoxicity Assay

Effect of ZSO or ZSE on RAW 264.7 or CRFK cells viability was evaluated using 3-(4,5-dimethylthiazol-2-yl)-2,5-diphenyltetrazolium bromide (MTT) assay [19]. Briefly, RAW 264.7 or CRFK cells were seeded in 96-well plates at a density of 2 × 10^5^ or 0.5 × 10^4^ viable cells per mL, respectively, in Dulbecco's modified Eagle's medium (DMEM, Gibco, Grand Island, NY, USA) containing 10% heat-inactivated fetal bovine serum (FBS, Gibco) and 1% penicillin-streptomycin (PS, Gibco). Cells were incubated at 37°C with 5% CO_2_ for 24 h, after which media were removed. ZSO or ZSE were dissolved in dimethyl sulfoxide (DMSO, Sigma, St. Louis, MO, USA), filtered through a 0.22 *μ*m filter (Millipore, Billerica, MA, USA), and added to each well at the indicated concentrations (0.0001–0.01% ZSO or 0.00001–0.001% ZSE). The plates were further incubated for 72 h at 37°C and 5% CO_2_. Then, 10 *μ*L of MTT (Gibco) solution was added to each well, followed by incubation at 37°C for 2 h. After removal of supernatant, 100 *μ*L of DMSO was added and incubated for 30 min. The absorbance at 570 nm was determined using a microplate reader (SpectraMax M2, Molecular Devices Corp., USA). The percentage of cell viability after treatment with ZSO or ZSE was calculated as follows: % cell viability = (Abs_treatment_/Abs_control_) × 100. All determinations were performed in triplicate. Cytotoxicity of DMSO was separately analyzed as described above. DMSO at a concentration of 0.01% showed >87% viability against RAW 264.7 or CRFK cells. Relative cell viability was analyzed based on DMSO control.

### 2.4. Cytopathic Effect (CPE) Assay

For CPE assay of ZEO or ZSO, cell viability was quantitatively determined using MTT assay [20]. RAW 264.7 and CRFK cells were seeded in 96-well plates (2 × 10^5^ cells and 0.5 × 10^4^ cells per well, resp.) in DMEM containing 10% FBS and 1% PS. After incubation for 12 h, media were removed and 10 *μ*L of ZSO (0.0001%, 0.001%, and 0.01%) or ZSE (0.00001%, 0.0001%, and 0.001%) and 10 *μ*L of FCV-F9 or MNV-1 were added to cells containing 80 *μ*L of fresh media for 48–72 h. Then, 10 *μ*L of MTT solution was added to each well and incubated at 37°C for 2 h. After removal of supernatant, DMSO was added and incubated for 30 min. The absorbance at 570 nm was determined using a microplate reader. All determinations were performed in triplicate.

### 2.5. Plaque Assay

To evaluate the antiviral activity of ZSO against FCV-F9 and MNV-1, the effect of ZSO was evaluated at different time points during virus infection using plaque assay [21]. Pretreatment of cells and viruses with ZSO was carried out separately. Pretreatment of RAW 264.7 cells was conducted as follows: ZSO in DMEM containing 10% FBS and 1% PS was added to confluent monolayers of RAW 264.7 cells and incubated at 37°C in a CO_2_ incubator for 1 h with gentle shaking. After complete aspiration of cell media containing the ZSO, a 10-fold serial dilution of virus stock (2~3 log_10_ PFU/mL) prepared in DMEM containing 10% FBS and 1% PS was inoculated into each well. After viruses were adsorbed for 1 h at 37°C in a CO_2_ incubator and the inocula were removed, 1 mL of DMEM containing 1.5% agarose, 5% FBS, and 0.5% PS was added to each well. The plates were then incubated for 42 h at 37°C in a CO_2_ incubator, after which cells were stained with 0.5% crystal violet and the number of plaques counted. DMSO and 2-thiouridine (2TU) were used as negative and positive controls, respectively. Pretreatment of MNV-1 with ZSO was performed by mixing equal volumes of ZSO and MNV-1, followed by incubation at room temperature for 1 h. Ten-fold serial dilutions of virus stock (2~3 log_10_ PFU/mL) were then inoculated onto confluent RAW 264.7 cell monolayers for 1 h at 37°C in a CO_2_ incubator. After virus adsorption, the same procedure as that described for pretreatment of RAW 264.7 cells was carried out.

For cotreatment, the same procedure as that described for pretreatment of 264.7 cells was carried out, except that confluent RAW 264.7 cell monolayers were infected with 100 *μ*L of virus stock (2~3 log_10_ PFU/mL), which was simultaneously incubated with ZSO for 1 h at 37°C in a CO_2_ incubator. For posttreatment, inocula were completely removed after virus adsorption (2~3 log_10_ PFU/mL) to cells, which were incubated with ZSO for 1 h. The same procedure as that described for pretreatment of cells was then carried out. FCV-F9 plaque assay using CRFK cells was also performed following the method described for MNV-1 and RAW 264.7 cells. An effective concentration to reduce the 50% plaque number (EC_50_) was calculated by regression analysis of the dose-response curves generated from these data [22].

### 2.6. Preparation and Identification of Fatty Acid Methyl Esters

Fatty acid methyl esters (FAMEs) were prepared according to AOAC Official Method 969.33 [23]. Briefly, 500 mg of ZSO was placed in a boiling flask to which 4 mL of 0.5 M sodium hydroxide in methanol was added. The flask was refluxed for 10 min, after which 5 mL of 12.5% boron trifluoride (Sigma-Aldrich Co. LLC, St Louis, MO, USA) in methanol was added. After refluxing for 2 min, 5 mL of* n*-hexane was added, and the flask was again refluxed for 1 min. Saturated sodium chloride solutions were then added to the flask. The upper layer containing FAMEs was pipetted into a vial containing anhydrous sodium sulfate to remove water. The solution was finally filtered and used for subsequent gas chromatography/mass spectrometry (GC/MS) analysis. GC/MS (Agilent 6890 GC/5973 MSD) was used to analyze fatty acid composition with an Omegawax column (30 m length × 0.25 mm i.d. × 0.25 *μ*m film thickness; Sigma-Aldrich Co.). Injection volume was 1 *μ*L, and the flow rate of helium as a carrier gas was 5.0 mL/min. The oven temperature was held constant at 140°C for 5 min, increased to 240°C at a rate of 5°C/min, and then held constant at 240°C for an additional 20 min. The temperatures of the injector and detector were 250°C. The MS was operated in electron ionization mode at 70 eV, scanning masses from 33 to 330 m/z. Identification of peaks on the chromatogram was performed the same way as that described for essential oils.

### 2.7. Statistical Analysis

Experimental results are expressed as mean ± SD. Data were analyzed using ANOVA with SAS software (version 9.2, SAS Institute, Cary, NC, USA), and the means were separated with Duncan's multiple range test. Means with a value of *P* < 0.05 were considered statistically significant.

## 3. Results and Discussion

### 3.1. ZSE Do Not Inhibit FCV-F9 and MNV-1 Infectivity

We first evaluated the effect of ZSE on RAW 264.7 or CRFK cell viability. Cells were exposed to ZSE at concentrations of 0.00001%, 0.0001%, and 0.001% for 72 h. RAW 264.7 or CRFK cells exhibited >85% cell viability upon treatment with 0.001% ZSE (data not shown). Next, antiviral activity of ZSE was examined using CPE assay. We found that when FCV-F9 or MNV-1 was incubated with up to 0.001% ZSE for 48–72 h, inhibition of cytopathic effects on CRFK or RAW 264.7 cells could not be detected (data not shown). These results suggest that ZSE do not inactivate the foodborne viral surrogates FCV-F9 and MNV-1. In accordance with our results, previous reports have also shown that MNV-1 is unaffected by hyssop and marjoram essential oils at 0.02% [12]. Further, FCV has been shown to survive on inoculated baby-leaf salad during refrigerated storage for 9 days in the presence of clove or zataria essential oils at 10% concentration [11]. However, the titer of FCV was found to be significantly reduced with essential oils from oregano, clove, and zataria at 37°C [24]. Oregano essential oil and its primary active component, carvacrol, were shown to be effective against MNV [25]. In addition, several research groups have demonstrated antiviral activities of essential oils from tea tree, chamomile, and* Lantana grisebachii* against HSV-1, HSV-2, and dengue virus type 2, respectively [13–15]. The major constituents of essential oils from pericarp of* Z. schinifolium* are linalool, d-limonene, and sabinene [9]. In this study, the main compound of essential oils from seeds of* Z*.* schinifolium* was found to be estragole (42%), which has a sweet-herbaceous anise-fennel type odor (Table 2).

### 3.2. Protective Effect of ZSO against MNV-1 and FCV-F9 Infectivity

RAW 264.7 or CRFK cells showed >87% cell viability upon exposure to 0.01% ZSO. ZSO at a concentration of 0.01% also resulted in 33% and 52% inhibition against FCV-F9 and MNV-1, respectively, via CPE assay. Next, to assess the antiviral mechanism of action of ZSO against FCV-F9 and MNV-1, the antiviral effect of ZSO was examined at different time points during virus infection using plaque assay. Plaque assay can be used to target the multiplication cycle of the calicivirus attachment of the viral protein to the cellular receptor, internalization of virion into the cell, replication of the virus, and release of the mature virion from the cell [26]. Pretreatment can assess the ability to inhibit attachment of the virus to cells, and the co- and posttreatments can test inhibition of virus internalization and replication, respectively.

**Figure 1:**
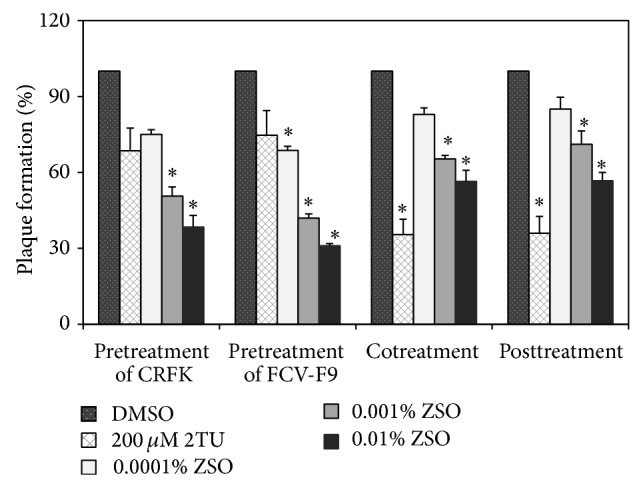
Antiviral activity of* Z*.* schinifolium* seed oils (ZSO) against FCV-F9 using plaque assay. ZSO were added at different time points during FCV-F9 infection of CRFK cells. Pretreatment of cells or virus represents incubation with ZSO for 1 h prior to viral infection; cotreatment represents simultaneous incubation of ZSO during viral infection for 1 h; posttreatment represents incubation for 1 h after viral infection to cells. Plaque numbers of ZSO-treated sample were measured in triplicate and compared with those of a DMSO-treated negative control. 2TU was used as a positive control. Within each treatment, asterisk denotes significant reduction of plaque formation relative to negative control (*P* < 0.05).

For FCV-F9, strong inhibition of FCV-F9 was achieved upon pretreatment with 0.01% ZSO in a dose-dependent manner, resulting in 70% inhibition (Figure 1), whereas 2TU as a positive control showed 25% inhibition at 200 *μ*M. Norovirus RNA-dependent RNA polymerase (RdRp) is involved in the synthesis of viral genomic RNA. RdRp, encoded by open reading frame 1 of the norovirus genome, is one of the key targets for the development of novel antiviral agents [27]. Ribavirin (1-*β*-D-ribofuranosyl-1,2,4-triazole-3-carboxamide) and 2TU, which are nucleoside analogs, inhibit viral replication by blocking the active site of RdRp [28]. In addition, 2TU has a stronger inhibitory effect on MNV-1 replication than ribavirin in RAW 264.7 cells [29]. In the present study, 2TU was used as a positive control. Pretreatment of CRFK cells with 0.01% ZSO resulted in 60% inhibition. The EC_50_ values of ZSO were 0.0007% and 0.001% upon pretreatment of FCV-F9 and CRFK cells, respectively. In comparison, co- and posttreatments with 0.01% ZSO resulted in 44 and 43% inhibition, respectively. These results suggest that the inhibitory activity of ZSO was exerted by direct interaction with FCV-F9 virion.

**Figure 2:**
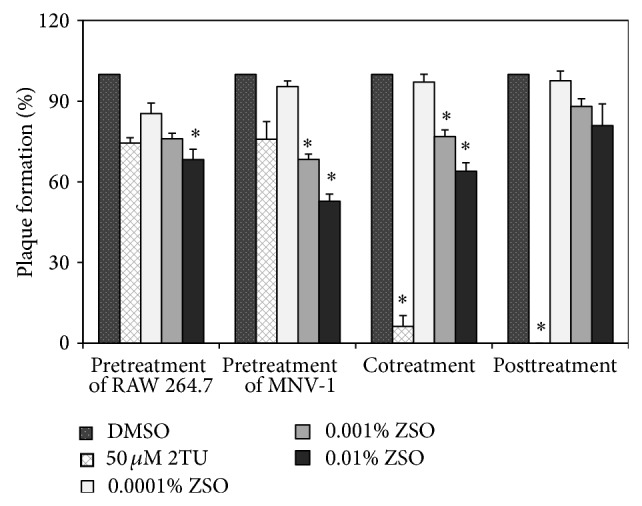
Antiviral activity of* Z*.* schinifolium* seed oils (ZSO) against MNV-1 using plaque assay. ZSO were added at different time points during MNV-1 infection of RAW 264.7 cells. Pretreatment of cells or virus represents incubation with ZSO for 1 h prior to viral infection; cotreatment represents simultaneous incubation of ZSO during viral infection for 1 h; posttreatment represents incubation for 1 h after viral infection to cells. Plaque numbers of ZSO-treated sample were measured in triplicate and compared with those of a DMSO-treated negative control. 2TU was used as a positive control. Within each treatment, asterisk denotes significant reduction of plaque formation relative to negative control (*P* < 0.05).

The effect of ZSO on another foodborne viral surrogate, MNV-1, was also examined using plaque assay. Upon pretreatment of MNV-1, ZSO showed a moderate inhibitory effect, reaching 47% at 0.01% concentration, whereas 2TU at 50 *μ*M resulted in 24% inhibition (Figure 2). Pretreatment of RAW 264.7 cells with ZSO exhibited 32% inhibition at 0.01%. When ZSO was added simultaneously to the cells with virus or the virus was posttreated with ZSO, plaque formation was inhibited by 36% and 20%, respectively, at a concentration of 0.01%. Likewise, these data suggest that the inhibitory activity of ZSO was exerted by direct interaction with MNV-1 virion.

The antiviral activities of ZSO against FCV-F9 and MNV-1 increased in a dose-dependent manner up to maximal antiviral activity upon pretreatment. Furthermore, FCV-F9 was more sensitive to ZSO than MNV-1, and EC_50_ of ZSO upon pretreatment with FCV-F9 was 0.0007%. Similar results have reported that FCV-F9 is more significantly inhibited by black raspberry juice or grape seed extract as compared with MNV-1 [21, 30]. In addition, MNV-1 is known to be more resistant to pH, heat [31], and environmental conditions [32] than FCV-F9. Taken together, our results suggest that ZSO can affect FCV-F9 and MNV-1 possibly by blocking virus attachment to host cells. It is therefore possible that ZSO can be used to control foodborne viral infection. Further studies are in progress to characterize active compounds of ZSO and their specific antiviral mechanisms against FCV-F9 and MNV-1.

### 3.3. Fatty Acid Composition of ZSO

As ZSO showed inhibitory activities against FCV-F9 and MNV-1, we analyzed the fatty acid composition of ZSO. The fatty acid methyl esters (FAMEs) of ZSO were prepared and analyzed by GC/MS. A total of eight fatty acids were identified, representing 98.31% of the total amount (Table 3). Oleic acid (35.36%), linoleic acid (22.6%), palmitic acid (18.5%), and linolenic acid (15.6%) were found to be the major fatty acids in ZSO, followed by palmitoleic acid (3.0%) and stearic acid (2.7%). In addition, arachidic acid and myristic acid were minor fatty acids. Among the identified fatty acids, unsaturated fatty acids constituted 76.6% of the oils.

## 4. Conclusions

In the present study, we determined the antiviral effects of oils (ZSO) and essential oils (ZSE) from seeds of* Z*.* schinifolium* against foodborne viral surrogates. ZSE, which comprised 42% estragole, showed no inhibitory effect against FCV-F9 or MNV-1. However, maximal antiviral effect was observed upon pretreatment of FCV-F9 or MNV-1 with ZSO, which contained oleic acid, linoleic acid, palmitic acid, and linolenic acid as the major fatty acids. These results suggest that the inhibitory activity of ZSO was exerted by direct interaction with FCV-F9 or MNV-1 virion. Therefore, ZSO may be a food material candidate for control of foodborne viral disease.

## Figures and Tables

**Table 1:** Preparation and yields of oils (ZSO) and essential oils (ZSE) from seeds of *Z*. *schinifolium*.

	Part of plant	Extraction method	Yield (v/w)
*Z*. *schinifolium* seed oils (ZSO)	Seed, dried	Extraction by oil press	80.1%
*Z*. *schinifolium* essential oils (ZSE)	Seed, dried	Hydrodistillation by Clevenger-type apparatus	0.13%

**Table 2:** Compounds of essential oils (ZSE) from seeds of *Z*.*  schinifolium* ^a^.

Compounds	RI^b^	Relative peak area (%)	Odor description^c^	Identification^d^
2-Nonenal	1150	0.12 ± 0.01	Fat, orris, and cucumber	RI, MS
Estragole	1200	42.01 ± 3.05	Licorice, anise	RI, MS
2,4-Decadienal	1320	4.87 ± 0.05	Seaweed	RI, MS
*α*-Cubebene	1354	0.12 ± 0.01	Herb, wax	RI, MS
2-Undecenal	1360	3.81 ± 0.05	Sweet	RI, MS
*β*-Elemene	1398	0.21 ± 0.01	Herb, wax, fresh	RI, MS
*β*-Caryophyllene	1430	0.11 ± 0.01	Wood, spice	RI, MS
*β*-Selinene	1436	0.05 ± 0.01	Herb	RI, MS
*α*-Humulene	1467	0.21 ± 0.04	Wood	RI, MS
*α*-Gurjunene	1458	0.18 ± 0.03	Wood, balsamic	RI, MS
Caryophyllene oxide	1598	0.21 ± 0.02	—	RI, MS
Spathulenol	1609	0.11 ± 0.01	Herb, fruit	RI, MS
Palmitic acid	1960	19.86 ± 1.27	—	RI, MS
Oleic acid	2061	20.97 ± 0.35	Fat	RI, MS

Total identified (%)		92.80		

^a^Mean ± SD (*n* = 3). GC was equipped with an HP-5MS column.

^b^Retention indices were determined using a series of alkanes C5–C30 as external references.

^c^Flavornet. Available at http://www.flavornet.org/flavornet.html. Accessed 2014 August 15.

^d^MS: mass spectrum; RI: retention index.

**Table 3:** Fatty acid composition of oils (ZSO) from seeds of *Z*.*  schinifolium* ^a^.

Fatty acid	RI^b^	Relative peak area (%)	Identification^c^
C14:0 myristic acid	1938	0.11 ± 0.05	MS
C16:0 palmitic acid	2162	18.51 ± 2.02	RI, MS
C16:1 palmitoleic acid	2208	2.99 ± 0.13	MS
C18:0 stearic acid	2430	2.67 ± 0.51	RI, MS
C18:1 oleic acid	2488	35.36 ± 3.60	RI, MS
C18:2 linoleic acid	2550	22.60 ± 0.88	MS
C18:3 linolenic acid	2608	15.63 ± 2.43	MS
C20:0 arachidic acid	2675	0.44 ± 0.16	MS

Unsaturated fatty acid (%)		76.58	
Saturated fatty acid (%)		21.73	
Total identified (%)		98.31	

^a^Mean ± SD (*n* = 3). GC was equipped with an Omegawax column.

^b^Retention indices were determined using a series of alkanes C5–C30 as external references.

^c^MS: mass spectrum; RI: retention index.
